# The Antiproliferative Effect of Cyclodipeptides from *Pseudomonas aeruginosa* PAO1 on HeLa Cells Involves Inhibition of Phosphorylation of Akt and S6k Kinases

**DOI:** 10.3390/molecules22061024

**Published:** 2017-06-20

**Authors:** Laura Hernández-Padilla, Dolores Vázquez-Rivera, Luis A. Sánchez-Briones, Alma L. Díaz-Pérez, José Moreno-Rodríguez, Mario A. Moreno-Eutimio, Victor Meza-Carmen, Homero Reyes-De la Cruz, Jesús Campos-García

**Affiliations:** 1 Laboratorio de Biotecnología Microbiana, Instituto de Investigaciones Químico-Biológicas, Universidad Michoacana de San Nicolás de Hidalgo, 58030 Morelia, Michoacán, Mexico; laura_190589@hotmail.com (L.H.-P.); dolores_vazquez1001@hotmail.com (D.V.-R.); biol.luis.22@gmail.com (L.A.S.-B.); aldiaz07@yahoo.com (A.L.D.-P.); victor_meza2004@yahoo.com.mx (V.M.-C.); 2 División de Investigación, Hospital Juárez de México, 07760 Ciudad de México, Mexico; jmoreno49@gmail.com (J.M.-R.); marioadan@inmunoquimica.com (M.A.M.-E.); 3 Laboratorio de Control Traduccional, Instituto de Investigaciones Químico-Biológicas, Universidad Michoacana de San Nicolás de Hidalgo, 58030 Morelia, Michoacán, Mexico; delacruz@umich.mx

**Keywords:** biomolecule, cyclodipeptides, antitumoral activity, cell proliferation, apoptosis, Akt–S6k signaling, HeLa

## Abstract

*Pseudomonas aeruginosa* PAO1, a potential pathogen of plants and animals, produces the cyclodipeptides cyclo(l-Pro-l-Tyr), cyclo(l-Pro-l-Phe), and cyclo(l-Pro-l-Val) (PAO1-CDPs), whose effects have been implicated in inhibition of human tumor cell line proliferation. Our purpose was to investigate in depth in the mechanisms of HeLa cell proliferation inhibition by the PAO1-CDPs. The results indicate that PAO1-CDPs, both purified individually and in mixtures, inhibited HeLa cell proliferation by arresting the cell cycle at the G0–G1 transition. The crude PAO1-CDPs mixture promoted cell death in HeLa cells in a dose-dependent manner, showing efficacy similar to that of isolated PAO1-CDPs (LD_50_ of 60–250 µM) and inducing apoptosis with EC_50_ between 0.6 and 3.0 µM. Moreover, PAO1-CDPs showed a higher proapoptotic activity (~10^3^–10^5^ fold) than their synthetic analogs did. Subsequently, the PAO1-CDPs affected mitochondrial membrane potential and induced apoptosis by caspase-9-dependent pathway. The mechanism of inhibition of cells proliferation in HeLa cells involves inhibition of phosphorylation of both Akt-S473 and S6k-T389 protein kinases, showing a cyclic behavior of their expression and phosphorylation in a time and concentration-dependent fashion. Taken together our findings indicate that PI3K–Akt–mTOR–S6k signaling pathway blockage is involved in the antiproliferative effect of the PAO1-CDPs.

## 1. Introduction

*Pseudomonas aeruginosa* colonizes several biological environments, such as soil, plants, and animal tissues, being an important opportunistic pathogen in humans, e.g., causing nosocomial infections [1,2]. Several mechanisms driving infection in the host have been attributed to the production of toxins, adhesins, siderophores, and a great number of virulence factors. Cyclodipeptides (CDPs) are cyclized molecules comprising two amino acids attached by peptide bonds; they are produced by a wide range of organisms, from bacteria to fungi to animals [3]. CDPs represent a new class of quorum-sensing (QS) signals, and they may act as interkingdom signals; nonetheless, their mechanism of action and physiological relevance are poorly understood [4].

CDPs are structurally diverse and have been implicated in multiple biological effects. The CDP cyclo(l-Phe-l-Pro) isolated from *Lactobacillus plantarum* has an antifungal effect [5], whereas CDPs cyclo(l-Leu-l-Pro), cyclo(l-Phe-l-Pro), cyclo(l-Val-l-Pro), cyclo(l-Trp-l-Pro), and cyclo(l-Leu-l-Val) isolated from the deep-sea bacterium *Streptomyces fungicidicus* show antifouling effects [6]. In *Staphylococcus aureus*, aureusimines A/B, namely, CDPs cyclo(l-Val-l-Tyr) and cyclo(l-Val-l-Phe), respectively, are involved in the regulation of bacterial virulence factors in a murine host [7]. In addition, it was reported that CDPs cyclo(l-Leu-l-Pro) and cis-cyclo(l-Phe-l-Pro) isolated from *Lactobacillus* show antiviral activity against the influenza A (H3N2) virus [8]. In mammalian cells, CDPs induce DNA damage via reactive oxygen species (ROS) [9]. Cyclo(l-Phe-l-His) of *Aspergillus ustus* inhibits the cell cycle in various cancer cell lines [10], whereas cyclo(l-Phe-l-Pro) from *L. plantarum* induces apoptosis in colon cancer HT-29 cells [11]. On the other hand, synthetic CDPs such as cyclo(Phe-Pro) induce apoptosis in the HT-29 colon cancer cell line, and cyclo(l-Cys-l-Leu) has a potential for scavenging of free radicals [12]. The molecular mechanisms behind the induction of cell death in cancer cell lines by CDPs involve biological processes such as microtubule polymerization [13]. Cyclo(d-Tyr-d-Phe) isolated from *Bacillus* sp. induces apoptosis via caspase 3 activation in the A549 pulmonary adenocarcinoma cell line [14]. In addition, our group has demonstrated that a crude mixture of CDPs obtained from the *P. aeruginosa* PAO1 strain, mainly composed of cyclo(l-Pro-l-Tyr), cyclo(l-Pro-l-Val), and cyclo(l-Pro-l-Phe), promotes cell death in cultured HeLa and Caco-2 cells, pointing to an apoptotic pathway as the mechanism underlying the inhibition of cell proliferation [15].

Cancer results from malfunction of fundamental cellular processes that control cell number, including cellular growth, proliferation, survival and metabolism. In this sense, oncogenic and tumor suppressor signals such as PI3K, Akt, Ras, Raf, TRK, NF1, LKN1, PTEN, p53, and TSC1 and TSC2 have largely involved [16,17]. The phosphatidylinositol 3-kinase (PI3K) signal transduction pathway has been studied extensively and is known to be involved in growth control and in diseases [16,17]. The mTOR kinase is a master regulator of cellular metabolism, acting downstream of a more complex cell signaling network. The mTOR kinase exists in two complexes: mTORC1, which has been implicated in almost all cellular processes, such as anabolic metabolism, proliferation, protein, lipid, and nucleotide synthesis, cell survival, cell mobilization, oxygen supply, energy, proliferative signals, and tumorigenesis, and blocks catabolic processes such as autophagy at the post-translational and transcriptional levels; while mTORC2 is involved mainly in actin cytoskeleton reorganization [18]. The mTORC1 pathway is frequently up-regulated in cancer, particularly under increased PI3K signaling due to oncogenic activation of PI3K or mutagenic inactivation of the lipid phosphatase PTEN [16].

Radiation and chemotherapy are the most common procedures for cancer therapy, however, serious collateral damage is associated with these methods. Hence, is necessary to find alternative and specific cancer treatments, and in this regard, the PI3K–Akt–mTOR signaling pathway has been suggested as a target for the design of molecules with anticancer pharmacological properties that could be used in the control and treatment of human diseases including cancer. In this sense, CDPs have been shown to have toxic effects on tumor cell lines via an Akt-dependent mechanism [19], but the evidence is scarce.

## 2. Results

### 2.1. Purified CDPs from P. aeruginosa PAO1 Affect HeLa Cells Viability

Quantification of CDPs in the supernatant of *P. aeruginosa* cultures was conducted previously, identifying CDPs cyclo(l-Pro-l-Tyr), cyclo(l-Pro-l-Val), and cyclo(l-Pro-l-Phe) by GC-MS, RMN-H, and RMN-C [20]. During subsequent studies, an additional CDP was identified, corresponding to cyclo(l-Pro-l-Leu), whose mass fragmentation patterns showed >96% probability with respect to the NIST library. Therefore, the CDP mixture from the PAO1 strain (PAO1-CDPs) was composed of following proportions: cyclo(l-Pro-l-Tyr) ~25%, cyclo(l-Pro-l-Val) ~25%, cyclo(l-Pro-l-Phe) ~30%, cyclo(l-Pro-l-Leu) ~10%, and other compounds ~10% (Figure S1, Supplementary Material).

As previously described, we used this crude mixture of CDPs isolated from *P. aeruginosa* PAO1 cultures to evaluate the antiproliferative effect on HeLa and Caco-2 cells [15]. Moreover, to determine whether the most abundant CDPs in the mixture have differential antiproliferative activities, a preparative CDP purification procedure was carried out. After separation and concentration of the fractions, CDP structures were confirmed by GC-MS. The purified fractions with ~85–95% purity corresponding to the three major CDPs were used to determine the effect on HeLa cell viability, by the MTT assay. Additionally, the response to CDPs of bacterial origin was compared with that of the respective synthetic CDPs.

**Figure 1:**
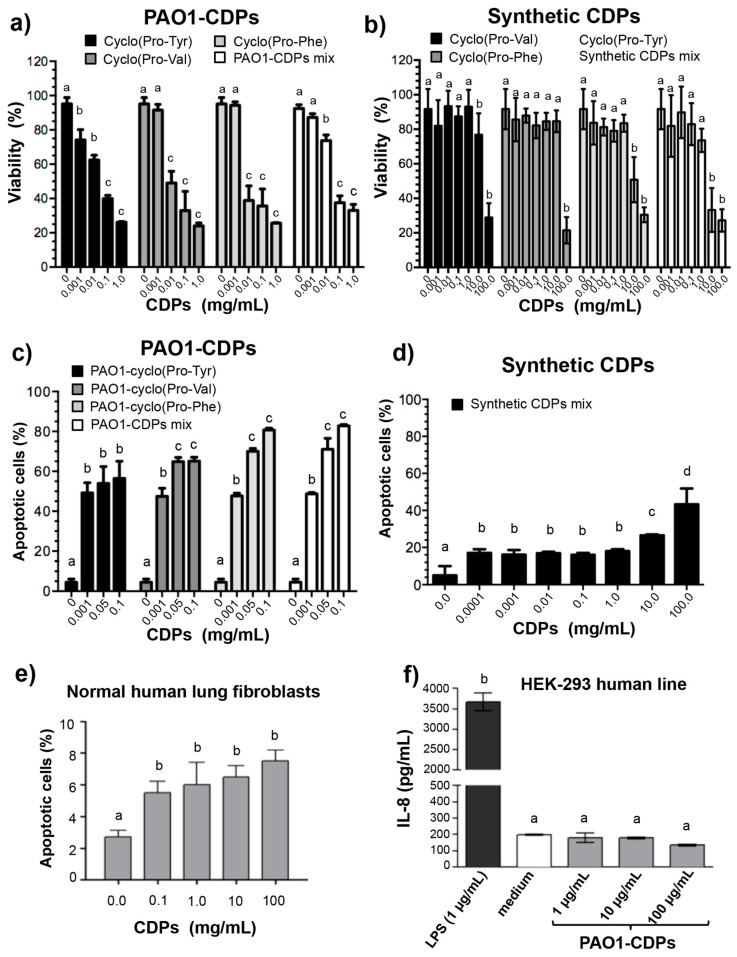
Effects of PAO1 and synthetic CDPs on HeLa cell viability and apoptosis induction. HeLa cells were incubated in CM medium and treated with CDPs for 24 h as described in Materials and Methods. Determination of viability of HeLa cells by an MTT assay treated with CDPs from *P. aeruginosa* PAO1 (**a**) and synthetic analogous CDPs (**b**). Induction of apoptosis in human HeLa cells by CDPs was analyzed in cultures grown in the CM medium and after treatment with CDPs for 24 h. Human cell lines were stained with annexin V and propidium iodide and analyzed by flow cytometry. The percentage of viability was determined by fluorescent cell quantitation in the dot plots, values were graphed as bars. Apoptosis induction in HeLa cells treated with PAO1-CDPs (**c)** and with the mixture of synthetic analogs of CDPs (**d**); (**e**) Apoptosis induction in normal human lung fibroblasts by the PAO1-CDP mixture at different concentrations determined as in (**c**); (**f**) IL-8 induction in HEK-293 cells by ELISA assay as described in Materials and Methods. Bars represent mean ± standard error (SE) of three independent experiments, *n* = 6. One-way analysis of variance (ANOVA) was carried out, with Tukey’s *post hoc* test; statistical significance (*p* < 0.01) of differences between treatments is indicated with different lowercase letters.

The results showed that PAO1-CDPs affected the viability of HeLa cells in a dose-dependent manner. Cell cultures showed 75% of dead cells after treatment with PAO1-CDPs, using either individual (purified) CDPs and the crude CDP mixture at 1.0 mg/mL after 24 h, being the cyclo(l-Pro-l-Phe) slightly more active against HeLa cells than the other CDPs (Figure 1a; Table 1). Moreover, the effect of synthetic CDPs on viability, separated or in the mixture, was significantly less active than PAO1-CDPs. At concentrations below 1.0 mg/mL, CDPs of bacterial origin showed HeLa cells viabilities below 20%, whereas the cell viability was ~80% with the synthetic CDPs at the same concentration (Figure 1b). For HeLa cells, the half-lethal dose (LD_50_) for the individual and purified PAO1-CDPs was in the concentration range of 0.015–0.06 mg/mL (60–250 μM) after 24 h of treatment. Interestingly, the LD_50_ for the crude PAO1-CDP mixture was in the same range as for the purified PAO1-CDPs (Table 1). On the other hand, LD_50_ of individual synthetic CDPs was between 2 and 150 mg/mL (10–600 mM); furthermore, the mixture of synthetic CDPs was more bioactive (5- to 50-fold) than the individual synthetic compounds (Table 1). Remarkably, the LD_50_ data indicate that the PAO1-CDPs were ~1000-fold more bioactive inducing cellular death than their synthetic analogs.

### 2.2. Purified CDPs from P. aeruginosa PAO1 Induce Apoptosis in HeLa Cells

To determine whether some of the CDPs that constitute the PAO1-CDPs mixture are the compounds responsible of the induction of apoptosis in HeLa cells, the effect of PAO1-CDPs separately or in mixtures was determined by flow cytometry using annexin V and propidium iodide as probes. The percentage of fluorescent cells (PFC), corresponding to cells that were positive for the annexin V marker was ≤5% for HeLa cells without CDPs (Figure 1c). On the other hand, cells treated with the purified PAO1-CDPs added separately or in mixtures, showed ~50% of apoptotic HeLa cells at 1.0 μg/mL (4 μM) after 12 h of treatment (Figure 1c); interestingly, for the synthetic CDP mixture (that was more active than its individual compounds), the apoptosis induction was ~18% at the same concentration (Figure 1d). These results indicate that after 12 h of treatment, the PAO1-CDPs mixture induces apoptosis in ≥95% of HeLa cells, showing a half-effective concentration (EC_50_) between ~1.6 × 10^−4^ and 5 × 10^−4^ mg/mL (~0.6–3 μM). However, no significant differences between isolated PAO1-CDPs and the crude PAO1-CDP mixture were observed, independently whether CM or SS mediums were used (Figure 1c; Table 1). Conversely, for the synthetic-CDP mixture, the EC_50_ values for apoptosis induction were higher than 100 mg/mL (400 mM; Table 1). These data indicate that PAO1-CDPs were at least three log units more active for apoptosis induction in HeLa cells than their synthetic analogs.

On the other hand, the crude PAO1-CDP mixture produced a slight apoptosis induction (~8%) in normal human lung fibroblasts, but at the highest concentration tested 100 mg/mL (400 mM); Figure 1e). Additionally, to rule out the possibility that the apoptotic effect caused by the crude PAO1-CDP mixture is due to the presence of lipopolysaccharides (LPS) as additional compounds of bacterial origin, the interleukin IL-8 presence was determined in the HEK-293 human cellular line. The data showed that the crude PAO1-CDP mixture obtained as described our procedure does not induce IL-8 production in the HEK-293 cell culture at concentrations over 0.5 mM (Figure 1f). These results indicate that the crude PAO1-CDP mixture does not contain LPS at concentrations that can to induce the biological response observed, and hence, the apoptotic response provoked in HeLa cells are indeed due to PAO1-CDPs.

**Figure 2:**
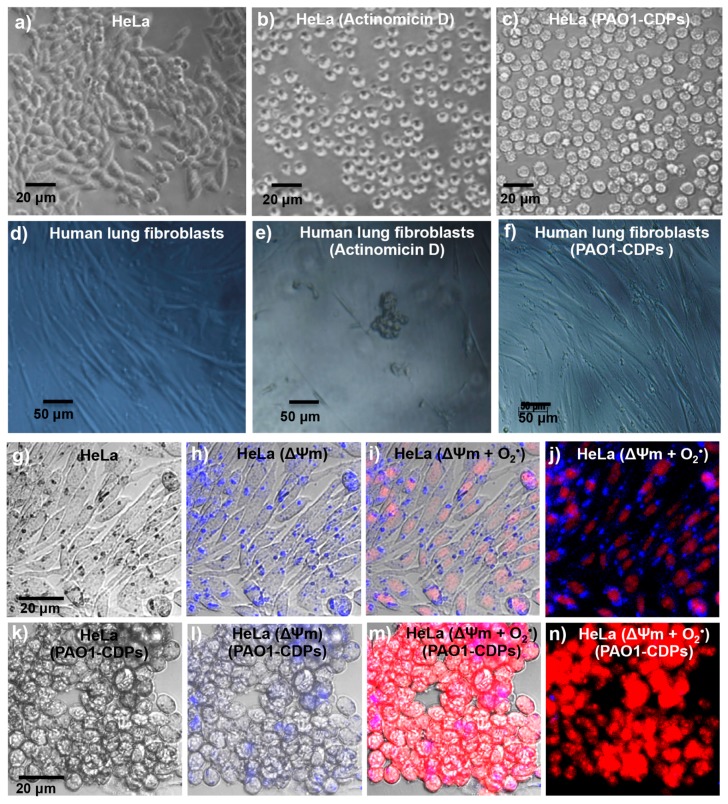
Morphological changes in human cell cultures stimulated with the CDP mixture from *P. aeruginosa* PAO1. (**a**–**n**) Images of human cells taken by means of phase contrast and confocal microscopy after treatment; (**a**–**c**) HeLa cell line; (**d**–**f**) Human lung fibroblast cell line. (**a**,**d**) DMSO (0.05%; negative control); (**b**,**e)** Actinomycin D (50 mg/mL; positive apoptosis control); (**c**,**f**) PAO1-CDP mixture (10 mg/mL) treatment for 2 h; (**g**) HeLa cells without treatment; (**h**) Determination of HeLa cells’ membrane potential (ΔΨm) using Rhodamine 123 without treatment. (**i**) HeLa cell membrane potential (ΔΨm) and superoxide (O_2_^•−^) quantification (using DHE probe) without treatment; (**j**) Same as in (**i**); but examination by dark field microscopy; (**k**–**n**) Conditions as in (**g**–**j**) but with treatment with the PAO1-CDP mixture. Images of the cells were taken at 40× magnification, using inverted phase-contrast microscope (HB0-50, Carl-Zeiss, San Diego, CA, USA) or confocal microscope (FV1000, Olympus, Center Valley, PA, USA).

In addition, the apoptosis induction in HeLa cells was confirmed by microscopic examination; the cells treated with the crude PAO1-CDP mixture at 0.01 mg/mL (40 μM) after 4 h of incubation, showed apoptotic cell morphology similar to the morphology of the cells treated with actinomycin D (10 mg/mL). Of note, apoptotic cell morphology was not observed in the normal human lung fibroblast line treated with high concentrations of the PAO1-CDP mixture (100 μM, Figure 2f).

### 2.3. CDPs from P. aeruginosa PAO1 Induce Apoptosis in HeLa Cells though a Caspase-9- and Caspase-3-Dependent Pathway

**Figure 3:**
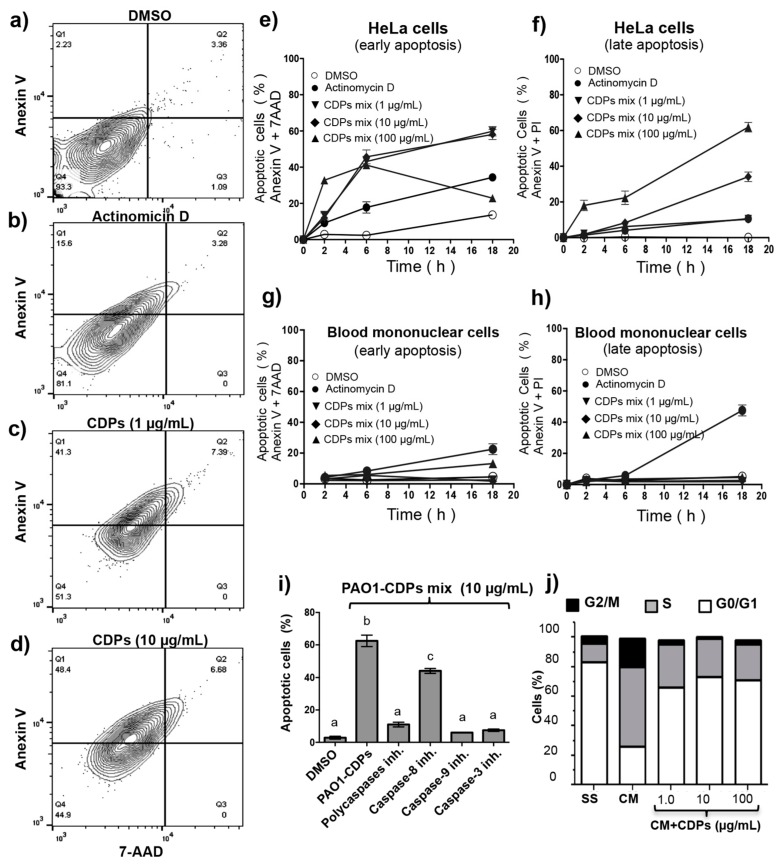
Apoptosis induction in HeLa cells by CDPs from *P. aeruginosa* PAO1. HeLa cells were incubated in the CM medium after treatment with PAO1-CDPs (various doses) as a function of time. Cells were stained with annexin V, 7-AAD, or propidium iodide and analyzed by flow cytometry. (**a**–**d**) The percentage of fluorescent cells determined in the dot plots is shown, corresponding to HeLa cells treated for 6 h with (**a**) DMSO; (**b**) actinomycin D (50 mg/mL), (**c**) PAO1-CDP mixture (1 μg/mL); (**d**) PAO1-CDP mixture (10 μg/mL). Q1, early apoptosis; Q2, late apoptosis; Q3, necrotic cells; Q4, viable cells; (**e**,**f**) Kinetic of induction early and late apoptosis stages in HeLa cells treated with the PAO1-CDP mixture; (**g**,**h**) Kinetics of induction of early and late apoptosis stages in blood mononuclear human cells treated with the PAO1-CDP mixture. Points of the plots represent mean ± standard error (SE) of three independent experiments; (**i**) Effects of inhibitors of apoptosis on HeLa cells in the presence of the PAO1-CDP mixture. The cells were incubated in the CM medium after treatment with 10 μg/mL PAO1-CDP mixture with the addition of caspases inhibitors. Cells were revealed with annexin V and analyzed by flow cytometry. The percentage of fluorescent cells (apoptotic cells) after each treatment was determined by means of the dot plots and is shown as bars in the graph. The inhibitors tested are indicated on the X-axis. Bars of the plots represent mean ± SE of three independent experiments, *n* = 3. One-way ANOVA was carried out, with Tukey’s *post hoc* test; statistical significance (*p* < 0.01) of differences between treatments is showed with different lowercase letters; (**j**) Effects of the PAO1-CDP mixture on the cell cycle in HeLa cells. HeLa cells were incubated in serum-free medium (SS) and serum-enriched medium (CM) after treatment with various doses of the PAO1-CDPs for 24 h. Cells were fixed with paraformaldehyde (4%) for 10 min on ice. Then, the cells were incubated with DAPI (1:1000) for 10 min at room temperature and analyzed by flow cytometry for DNA quantitation.

To identify the apoptotic pathway induced by the crude PAO1-CDP mixture, a more detailed study was carried out, determining apoptosis in HeLa cells at short response times. After 12 h of treatment with 6–60 μM of crude PAO1-CDP mixture, the cellular population was found mostly at early stages of apoptosis (~50% early with ~7% at late stages) (Figure 3a–d). Similar results were observed when tested in the concentration range of 1–100 μg/mL (6–600 μM; Figure 3e,f). Interestingly, cultures of normal human mononuclear blood cells treated with the crude PAO1-CDP mixture show minimal effect on apoptosis induction at early or late stages, with less than 10% of cell population at concentrations sufficient for apoptosis induction of ≥95% of HeLa cells (100 μg/mL; Figure 3g,h). These results indicate that the antiproliferative effect of the PAO1-CDP mixture in the HeLa cells was mediated by an apoptotic pathway instead of necrosis, indicating the participation of caspases as the molecular mechanism of cell death involved. To further elucidate this fact, the annexin V apoptosis-marker was used in the presence of caspase inhibitors. Data show that, although apoptosis was diminished (~20%) in the presence of the caspase-8 inhibitor (Z-IETD-FMK), it was strongly inhibited in HeLa cells treated with PAO1-CDPs plus the polycaspase inhibitor (Z-VAD-FMK), caspase-3 (Z-DEVD-FMK), or caspase-9 (Z-LEHTD-FMK) inhibitors (Figure 3i), indicating that the PAO1-CDPs mixture induces apoptosis mainly by the caspase-9- and caspase-3-dependent pathway (intrinsic pathway).

### 2.4. CDPs from P. aeruginosa PAO1 Arrest HeLa cells at the G0–G1 Transition

The PAO1-CDPs antiproliferative mechanism was further explored by determining cell distribution in different phases of the cell cycle, through measuring the intracellular DNA content by flow cytometry. HeLa cells growing in a CM medium (medium with serum) showed ~25% of cells population at the G0–G1 stage, ~20% of cells at the G2–M stage, and ~55% of cells in the S phase.

Comparatively, cell cultures treated with the crude PAO1-CDP mixture (because no significant differences were observed in comparison with purified PAO1-CDPs) at 6–600 μM showed cell populations ~70% of cells at the G0–G1 stage, ~3% of cells at the G2–M stage, and ~25% of cells in the S phase (Figure 3j). Additionally, an arresting culture condition of HeLa cells such as SS medium (medium without serum by at least 4 h), showed proportions of cell populations similar to PAO1-CDPs treatment. These results suggest that the PAO1-CDPs diminish the proliferation of HeLa cells, blocking the DNA synthesis (S phase) and arresting the cultures at the G0–G1 stage.

### 2.5. CDPs from P. aeruginosa PAO1 Affect Membrane Potential and Induce Superoxide in HeLa Cells

**Figure 4:**
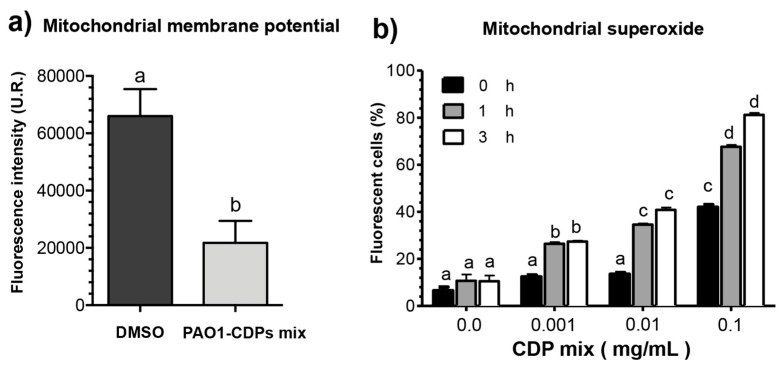
Membrane potential and superoxide quantification in HeLa cells treated with the CDP mixture from *P. aeruginosa* PAO1. Cell cultures were grown in the CM medium, harvested, and incubated in the CM or SS medium for 2 h. After that, the cell suspensions were incubated with or without PAO1-CDPs for 1 h at 37 °C. At the indicated time points, samples (100 μL) were resuspended in PBS and mixed with the Rhodamine 123 and DHE probes for quantitation of mitochondrial membrane potential and superoxide, respectively. The samples were incubated for 30 min and washed, and fluorescence was measured in real-time by flow cytometry. (**a**) Mitochondrial membrane potential and (**b**) superoxide quantification as percentage of fluorescent cells. Values are the mean of three independent experiments with 20,000 cells counted by flow cytometry per data point. SEM values are indicated as bars (*n* = 3), one-way ANOVA with Tukey’s *post hoc* test, significant differences (*p* < 0.01) are indicated by different lowercase letters.

Some toxic effects caused by CDPs in cell lines have been found to be associated with an increase in oxidative stress [9], or conversely, with beneficial effects of ROS scavenging [12]. Nevertheless, the mechanisms involved in ROS generation, accumulation, and type of ROS species generated by the CDPs are poorly known. So far, the findings indicate that the PAO1-CDP mixture induces apoptosis through an intrinsic pathway dependent on caspase-9 and caspase-3 activation, affecting the mitochondrial functionality prior to cytochrome *c* release, and therefore increased ROS generation is expected. The apoptotic pathway was confirmed by determining mitochondrial membrane potential (ΔΨm) in cell cultures using the fluorescent marker Rhodamine 123. The findings showed that the fluorescence intensity of cells decreased ~70% after treatment with the crude PAO1-CDP mixture in comparison with untreated cells (Figure 4a). Real-time ROS quantification by flow cytometry and examination by confocal microscopy were carried out using the DHE fluorescent probe, which mainly identifies the mitochondrial superoxide radical. As described above, the crude PAO1-CDP mixture was able to decrease 80% cell survival at 60–250 μM; thus, this concentration range was used for superoxide determination in HeLa cells growing in CM or SS medium. The percentage of fluorescent cells (PFC) was increased in HeLa cells in a PAO1-CDP concentration-dependent and treatment time-dependent manner (Figure 4b). Cell suspensions without treatment showed PFC of ~10%, whereas with the PAO1-CDP mixture, the PFC increased significantly to 30–80% after 3 h of treatment (Figure 4b).

These results indicate that the crude PAO1-CDP mixture increased superoxide generation in HeLa cells in the same fashion, suggesting that the mechanism of antiproliferative effects (toxicity) could be related with ROS generation events, impairing mitochondrial functionality (such as ΔΨm).

### 2.6. CDPs from P. aeruginosa PAO1 Modify the Akt and S6k Phosphorylation in HeLa Cells

Early studies on cell death regulation dependent on the caspase-9 protein have revealed the participation of Akt and small G protein p21-Ras kinases [21], and the PI3K–Akt–mTOR signaling pathway dysregulation has been extensively associated with cancer [22,23]. In this sense, our findings showed that the apoptotic caspase-9-dependent intrinsic pathway is involved in the antiproliferative effect of the PAO1-CDPs, and that HeLa cells are arrested in G0-G1 stages of the cell cycle. Accordingly, the participation of the PI3K–Akt–mTOR signaling pathway was analyzed next.

**Figure 5:**
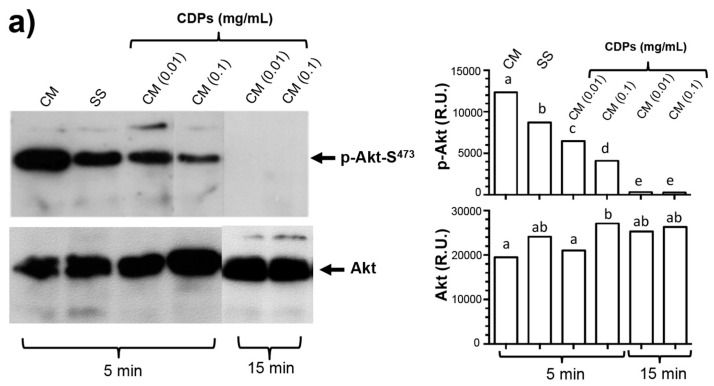

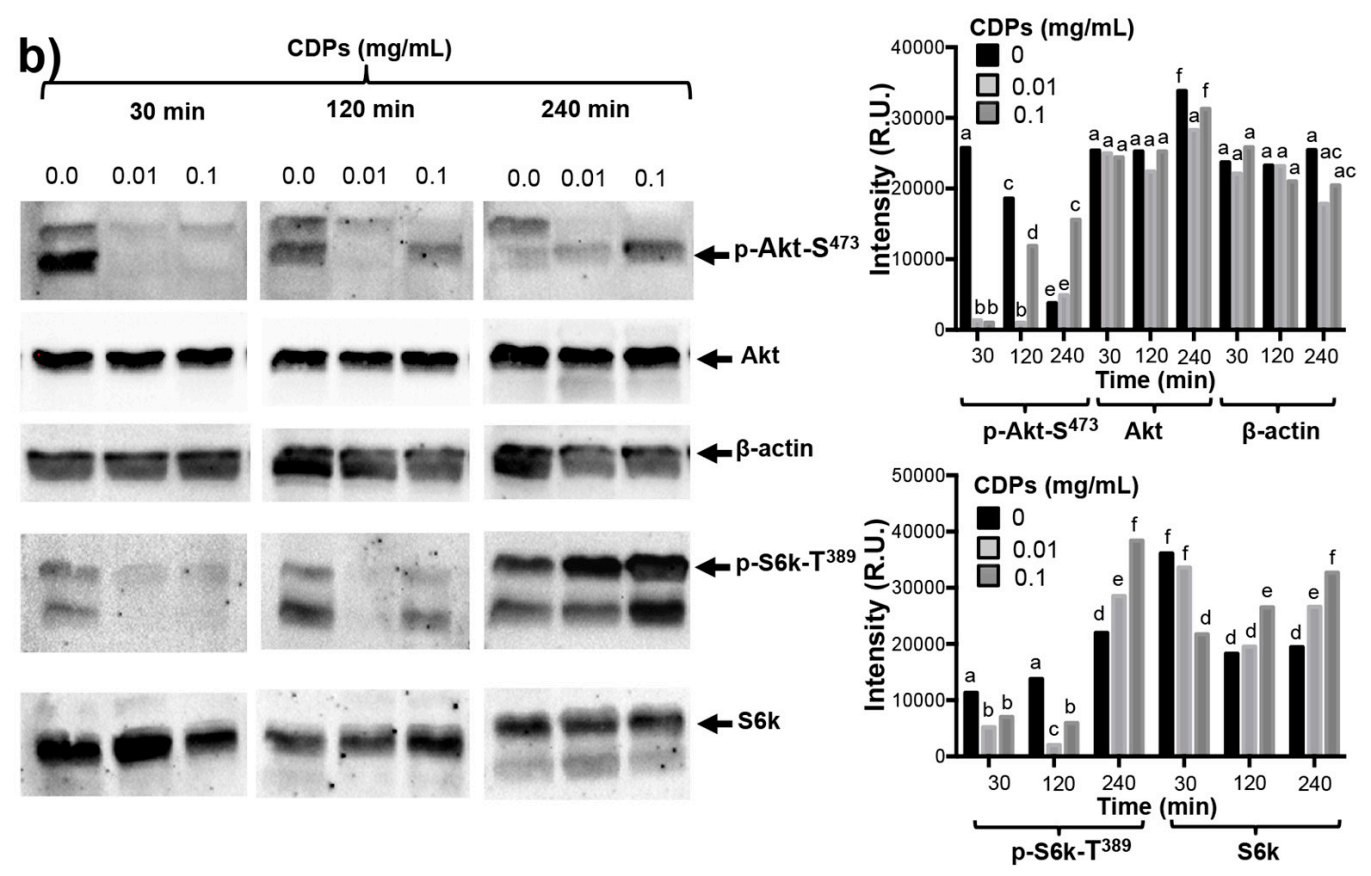
Effects of the CDP mixture from *P. aeruginosa* PAO1 on Akt and S6k phosphorylation and expression in HeLa cells. HeLa cells were incubated in SS and CM media and treated with 0.01 or 0.1 μg/mL PAO1-CDP mixture. At the indicated time points, cells were harvested and disrupted by sonication, and the solubilized proteins were separated by denaturing polyacrylamide gel electrophoresis (SDS-PAGE). Gels were electroblotted to PVDF membranes, and protein bands immunodetected using the indicated antibodies [anti-Akt (C-20-R), anti-Akt-phosphoryled 1/2/3 (Ser 473-R), anti-p70 S6 kinase α (H-160), anti-phosphoryled-p70 S6 kinase α (Thr 389)-R, and anti-β-actin] as the first antibody and a horseradish peroxidase (HRP)-conjugated goat anti-rabbit IgG antibody as the second antibody. Images correspond to representative gels from at least three independent treatments (left). Data correspond to the mean of three independent assays; the band intensity was determined by densitometry using the Image J software (right). (**a**) Immunodetection using the anti-phosphorylated Akt-S473 and anti-Akt antibodies after 5 and 15 min of treatment with the PAO1-CDP mixture; (**b**) HeLa cells extracts were obtained of cultures grown in CM media and treated with 0.01 or 0.1 mg/mL PAO1-CDP mixture. The same membranes reveled with anti-phosphorylated Akt-S473 were after immunodetected with the next antibodies: anti-Akt, anti-S6k, anti-phosphorylated-S6k-T389 and anti-β-actin. The assay was repeated at least three times using cell extracts from different cultures and treatments. A representative immunodetection assay is shown, and their plots of band intensity quantitation are shown to the right of the images. Bars represent mean of three densitometry determinations. Two-ways ANOVA was carried out, with Tukey’s *post hoc* test; statistical significance (*p* < 0.05) of differences between treatments is indicated with different lowercase letters.

As described above, the results showed that the PAO1-CDP mixture induces apoptosis efficiently at short times; thus, analysis of Akt protein expression and phosphorylation was conducted by immunodetection procedures. The results showed that the phosphorylation of the Akt-S473 protein (p-Akt-S473) was decreased in the HeLa cells treated with 0.01 and 0.1 mg/mL (40 and 400 μM) of the crude PAO1-CDP mixture at 5 min of treatment, and was totally undetectable after 15 min (Figure 5a). On the other hand, the total Akt protein showed a strong level of expression with significant difference at 0.1 mg/mL by 5 min, but without significant differences at concentration (0.01 mg/mL) (Figure 5a). At longer periods of PAO1-CDPs treatment (30–240 min), the phosphorylation of p-Akt-S473 was induced again in function of time and PAO1-CDPs concentration, while total Akt protein and β-actin (protein load control) remained at high levels and without significant changes between treatments (Figure 5b).

The S6K (S6 ribosomal protein kinase) is one of the downstream targets of Akt protein in the PI3K–Akt–mTOR signaling pathway; thus, the effect of PAO1-CDPs on the S6K protein phosphorylation was determined in HeLa cells. We found that the phosphorylated S6K-T389 protein (p-S6K-T389) also was strongly inhibited after short periods of the PAO1-CDP treatment (≤30 min), whereas induction of p-S6K-T389 amount was detected after longer periods (≥120–240 min); however, total S6K protein expression was not modified at 30 min, but it showed an increased induction at 60 and 120 min post CDPs treatment (Figure 5b). These results showed that the crude CDP mixture from *P. aeruginosa* PAO1 modify the phosphorylation of Akt-S473 and S6K-T389 proteins, as well as their expression levels in a time- and concentration-dependent manner.

## 3. Discussion

The quest for novel molecules with properties related to inhibition of cancerous cell growth is a scientific field of major interest. Natural molecules with antiproliferative activity are considered more target-specific than their synthetic analogs. Besides, peptides constitute a diverse family of natural compounds that also have been implicated in diverse biological functions. Cyclic peptides or their derivatives diketopiperazines of microbial origin are believed to have a strong pharmaceutical potential as antimicrobial and antifungal agents, immunomodulators, antioxidants, or anticancer agents [13,24]. CDPs possess intrinsic physiological advantages over other molecules, for example, chemical and enzymatic stability and structural and conformational specificity. These properties make them more promising than their non-CDP counterparts. Several approaches to CDP synthesis have been explored to discover synthetic analog molecules that can serve as novel drugs. Although CDPs have been discovered a long time ago and have been studied all this time, only recently have they aroused some interest because of their antiproliferative effects on cancerous cell lines [11,12,14,15].

*P. aeruginosa* is a pathogenic and opportunistic bacterium that produces a large number of virulence factors. CDPs can be considered the molecules that can regulate the production of virulence factors in a QS-dependent manner in this microorganism [25,26,27,28]. In the context of antiproliferative properties attributed to CDPs, we recently reported that a mixture of CDPs composed of cyclo(l-Pro-l-Tyr), cyclo(l-Pro-l-Val), and cyclo(l-Pro-l-Phe) isolated from the *P. aeruginosa* PAO1 strain can inhibit the proliferation of human tumor cell lines: HeLa and CaCo-2 [15]. In the present work, a CDP purification process was carried out with the aim to determine whether the antiproliferative effect previously observed in tumor cells are induced by some of the CDPs that constitute the PAO1-CDPs mixture or whether synergistic effects exit. We found that the CDP mixture from the PAO1 strain contains mainly the CDPs cyclo(l-Pro-l-Tyr), cyclo(l-Pro-l-Val), and cyclo(l-Pro-l-Phe) (≥80%) (Figure S1, Supplementary Material). When the effect of these isolated PAO1-CDP fractions on the viability or apoptosis of HeLa cells was tested, no significant differences were observed between them, except a slightly increased effect of cyclo(l-Pro-l-Phe) against HeLa cell viability than the other CDPs, but no over apoptosis induction (Figure 1a; Table 1). Furthermore, CDP synthetic analogs, though able to affect cell viability and to induce apoptosis, required a ~1000-fold higher concentration than PAO1-CDPs did. The LD_50_ of PAO1-CDPs was in the range between 0.010–0.03 mg/mL (60 and 250 μM) for HeLa cells; whereas this LD_50_ for the synthetic CDPs was between 10 and 400 mM (Table 1). Interestingly, we found that the PAO1-CDP mixture showed minimal apoptosis induction in blood mononuclear human cells cultures (<8%) at high concentrations such as 100 mg/mL (400 mM) and also in normal human lung fibroblasts cultures (<10%) at concentrations 100 µg/mL (4 µM) (Figure 1e and Figure 3g–h). In addition, we previously have been reported that a crude PAO1-CDP mixture showed IC_50_ of 0.53 mg/mL [15], however in this work the LD_50_ dose was of 0.06 mg/mL; this discrepancy is attributed to a better extraction process that let us to eliminate compounds such as AHL, LPS, or pigments.

Previously, researchers have described the growth inhibition of colon cancer HT-29, HeLa, and MCF-7 cells in culture by seven synthetic proline-based CDPs, revealing that cyclo(Phe-Pro) causes growth inhibition at 10 mM and induction of apoptosis (15% cells population) at 5 mM after 72 h of treatment [11,12]. In agreement with these data, we observed an inhibitory effect of viability and apoptosis induction at the same concentration (10 mM) with the same synthetic CDPs in HeLa cells (Figure 1). However, PAO1-CDPs showed highest antiproliferative activity than synthetic CDPs such as apoptosis induction at 0.6 μM after 12 h of treatment (Table 1), whereas for the synthetic mixture, it was observed at 400 mM. Furthermore, these data showed that PAO1-CDPs were at least three log units more active than their synthetic analogs. The probable reason for the observed effects is that molecules isolated from living entities such as *P. aeruginosa* are produced with chiral specificity, ensuring stereochemical specificity and therefore strong activity, as described elsewhere [11].

The antiproliferative mechanism of the PAO1-CDP mixture was explored further by determining cell population distribution in different phases of cell cycle. The results indicate that proliferation of the HeLa cells was arrested at the G0–G1 stage and at the DNA synthesis stage (S phase; Figure 3j). Quantification of apoptotic cells indicates that PAO1-CDPs caused apoptosis in HeLa cells mostly at early apoptotic steps. This effect was not observed in normal blood mononuclear cells (Figure 3g). Furthermore, the utilization of caspase inhibitors allowed us to determine that the induction of apoptosis in HeLa cells was dependent of the caspase-9 and -3 pathway (Figure 3i). This pathway was verified by measuring ΔΨm in cell cultures, confirming that the intrinsic apoptotic pathway was implicated. In line with this finding, HeLa cells treated with the crude PAO1-CDP mixture showed an increase in superoxide generation in a dose-dependent manner, confirming that the mechanism of cellular death caused by PAO1-CDPs also involves ROS generation, with superoxide being one of the major produced and accumulated species (Figure 4). The cells showed increased superoxide levels at the same times as the early stages of apoptosis induction with the PAO1-CDPs treatment, indicating that the strong ROS production occurs simultaneously with apoptotic events (Figure 3e and Figure 4b).

Dysregulation of the PI3K–Akt–mTOR signal transduction pathway has been shown to be associated with some carcinomas and has been implicated in the apoptotic intrinsic pathway too; this pathway performs essential functions in cellular growth regulation [29]. Additionally, the regulation of the apoptotic intrinsic pathway involves caspase-9 and subsequent cytochrome *c* proteolysis, where phosphorylation of pro-caspase-9 is related to the Akt protein kinase [21]. In this context, studies have revealed that the possible pathway for apoptosis induction by the CDPs cyclo(prolyl-tyrosyl) and cyclo(prolyl-phenylalanyl) isolated from *Bacillus* sp., is associated with Akt phosphorylation (inhibition to ~3–18% with respect to untreated cell cultures) [19]. Nonetheless, those authors did not present sufficient evidence to implicate these CDPs in the mechanism of apoptosis induction. In this sense, our results clearly show that the PAO1-CDP mixture was able to abrogate phosphorylation of both Akt-S473 and S6k-T389 protein kinases in a time- and concentration-dependent manner in HeLa cells in short time periods (5–30 min) (Figure 5). Additionally, we found that the phosphorylation and dephosphorylation of Akt-S473 and S6k-T389 protein kinases showed a cyclic behavior in HeLa cells: after inhibition of phosphorylation by PAO1-CDPs treatment, phosphorylation of both proteins was detected again, but after longer periods of time (120–240 min). We also determined the phosphorylation of Akt-S473 protein in free cell extracts of HeLa cultures treated with the crude PAO1-CDP mixture at prolonged times (12–48 h), but it was impossible to detect the p-Akt-S473 or Akt protein isoforms, observing massive protein degradation on the SDS-PAGE gels.

Akt phosphorylation and dephosphorylation have been reported in HeLa cells subjected to serum starvation in a cyclic biphasic behavior. Incubation periods less than 12 h led to low levels of Akt-S473 phosphorylation, but after periods longer than 12 h, higher levels of p-Akt were observed involving endogenous insulinlike growth factor (IGF) synthesis under deficient culture conditions, such as serum deprivation [30]. It is interesting whether CDPs themselves also induce endogenous synthesis of molecules that can activate the Akt pathway, and more experiments are needed to explain this biphasic behavior of Akt phosphorylation.

mTORC1 controls the rate of protein synthesis through phosphorylation and activation of the S6k protein kinase and eukaryotic translation initiation factor 4E (eIF4E)-binding protein 1 (4E-BP1), promoting mRNA translation and protein synthesis [23]. In general, our findings confirm that PAO1-CDPs are capable of inducing apoptosis in human tumor HeLa cells involving the inhibition of Akt phosphorylation and subsequently the phosphorylation of the downstream S6k protein target. Because cell proliferation is associated with p-Akt/p-S6k levels, our findings suggest that the inactivation of the TORC1 complex probably participates in the antiproliferative effect of the PAO1-CDPs in HeLa cells, thereby pointing to inactivation of the PI3K–Akt–mTOR signal transduction pathway as PAO1-CDPs’ mechanism of action.

Akt regulates metabolism, survival, apoptosis, growth, and proliferation, whereas mTORC2 directly activates Akt by phosphorylating its hydrophobic motif (Ser473), a site required for its maximal activation [16,31]. Hence, the cyclic phosphorylation behavior of Akt-S473 observed during PAO1-CDP treatment of HeLa cells suggests that mTORC2 activity may be involved. The mTORC2-dependent Akt phosphorylation leads to activation of mTORC1; thus, mTORC2 may indirectly suppress autophagy [18,32].

The heterodimer consisting of tuberous sclerosis 1 (TSC1; also known as hamartin) and TSC2 (also known as tuberin) is a key upstream regulator of mTORC1 and functions as a GTPase-activating protein (GAP) for Ras homolog enriched in brain (Rheb) GTPase. The GTP-bound form of Rheb directly interacts with mTORC1 and strongly stimulates its kinase activity. As a Rheb GAP, TSC1–2 heterodimer negatively regulates mTORC1 by switching Rheb to its inactive GDP-bound state [16]. Phosphorylated Akt disrupts the heterodimer by phosphorylating TSC1, thereby abrogating its GAP action on Rheb, leading to mTORC1 activation, thus promoting cell proliferation and inhibiting autophagy.

TSC1 or TSC2 dysfunction is also implicated in uncontrolled growth and cancer [18]. In contrast, low cellular energy levels or hypoxia induce TSC1/2 heterodimer formation inhibiting mTORC1 activation. Autophagy is a cellular process necessary for development and tissue homeostasis and participates in various physiological and pathologic processes (including exercise, metabolic adaptation, and disorders such as neurodegenerative diseases, infectious diseases, cardiovascular diseases, cancer, and aging) [18]. Because mTORC1 plays essential roles in autophagy, it is a potential pharmacological target. Therefore, identification of novel molecules with the capacity for modulation of autophagy via mTOR-dependent mechanisms is of great scientific interest in terms of treatment of human diseases.

## 4. Materials and Methods

### 4.1. Chemicals and Reagents

Dulbecco’s modified Eagle’s medium (DMEM), fetal bovine serum (FBS), antibiotic and antimycotic solution (100X) containing penicillin, streptomycin, amphotericin B, 4,6-diamidino-2-phenylindole (DAPI), 3-(4,5-dimethylthiazol-2-yl)-2,5-diphenyltetrazolium bromide (MTT) were purchased from Sigma-Aldrich Co. (St. Louis, MO, USA). Tissue-culture plasticware was acquired from Corning (New York, NY, USA), Alexa Fluor 488 Annexin V and the PI/dead cell apoptosis kits (Invitrogen, Life Technologies, Carlsbad, CA, USA), and synthetic CDPs cyclo(-Pro-Val), cyclo(-Pro-Tyr), and cyclo(-Phe-Pro) (G-4730, G-4715, and G-4720, respectively) were acquired from Bachem Co. (Torrance, CA, USA).

### 4.2. Bacterial Strains and Culture Conditions

The *P. aeruginosa* PAO1 wild type [33] was grown in Luria-Bertani (LB) broth at 37 °C, with shaking. Solid media were prepared by adding 1.5% (*w*/*v*) agar. Antibiotic concentrations used for the *P. aeruginosa* were 200 μg/mL streptomycin; all reagents were purchased from Sigma-Aldrich Co.

### 4.3. Solvent Extraction and Chemical Characterization of CDPs from the P. aeruginosa PAO1 Strain

A 2.5 × 10^8^ CFU inoculum of *P. aeruginosa* WT was placed in 300 mL of LB broth and incubated in a growth cabinet 24 h at 37 °C for bacterial growth. Cell-free supernatants were prepared by centrifugation (10,000× *g* at 25 °C by 10 min; in an 5810R centrifuge (Eppendorf Hauppauge, NY, USA). The resulting supernatant was extracted twice with two volumes of ethyl acetate supplied with acetic acid (0.1 mL/L). The extract was evaporated to dryness using a rotavapor (Buchi-210 Lab, Buchi, Flawil, Switzerland) at 60 °C under vacuum. The residue was solubilized in methanol–acetonitrile (1:1), the undissolved residue was removed by centrifugation and the sample was evaporated to dryness, and finally dissolved in DMSO–water (1:3) rendering the crude PAO1-CDPs mixture. Analysis of extracts was carried out using High Performance Liquid Chromatography (HPLC, model 240, Varian, Santa Clara, CA, USA) using a Photodiode Array detector (Varian 410) and a reverse-phase HPLC column Sephasil-Peptide C18, 12 µm, 4.6 mm × 250 mm (Amersham, Pittsburgh, PA, USA). Fractions were eluted with water-acetonitrile, starting with a equilibration solvent mix of 0:100; followed by a gradient linear up 60:40, at flow of 1 mL/min by 15 min, following with return to 0:100 solvent mix in 3 min and an equilibrium phase during 2 min. The deionized water and HPLC-grade acetonitrile were filtered and degasified (J.T. Baker, Center Valley, PA, USA). The extract was also analyzed for CDPs identification by gas chromatography-mass spectrometry (GC-MS, GC-6850 Series II equipped with a MS-5973, Agilent Technologies Inc., Santa Clara, CA, USA) as previously described [20]. Relative CDP proportions were determined by area units showed in chromatograms of the GC-MS analysis. For dose-response assays, the crude PAO1-CDPs mix was evaporated to dryness, weighed out, and dissolved with DMSO-water 1:3 to prepare a 100 mg/mL concentration as stock solution.

### 4.4. Cell Line Growth

The human cancer cell line HeLa was obtained from the American Type Culture Collection (ATCC, Manassas, VA, USA), HEK-293/MD2/CD14 cell line used in IL-8 induction assay (InvivoGene, San Diego, CA, USA), peripheral blood mononulcear cells (PCMB) obtained from healthy volunteers by isolation through Ficoll gradient and human lung fibroblast cells were kindly provided by Dr. Moises Selman and Dr. Adan Moreno (Hospital Juárez de México, México City). Cell procedures were performed under class II biological safety cabinets. Cells were cultured in DMEM supplemented with 10% (*v*/*v*) FBS (complete medium, CM), and 1% antibiotic (10,000 units of penicillin, 10 mg streptomycin, and 25 g of amphotericin B per mL, Sigma-Aldrich Co.) solution. The cultures were fed twice a week and maintained at 37 °C under 80% humidity and incubated in an atmosphere of 5% CO_2_. HeLa cells were collected by trypsinization using trypsin/EDTA buffered solution for 5 min at 30 °C, followed by the addition of serum-enriched complete medium (CM) to stop trypsin action. After trypsinization the cells were collected and washed with CM. Finally, cells were counted in a hemocytometer chamber and incubated in fresh CM media.

### 4.5. Cell Viability Assay

Cell viability was determined by the colorimetric method using MTT dye. Briefly, HeLa cells were seeded in 96-well flat-bottomed plates (Thermo Fisher Scientific, Grand Island, NY, USA) at a density of 3 × 10^4^ cells per well in 200 μL of CM and incubated by 24 h at 37 °C with 5% CO_2_ as described above. Then, the medium was removed and replaced with fresh CM or serum-free medium (SS). Then, cells were incubated with the CDPs solution at indicated concentrations. Cells were incubated for another 24 h at 37 °C with 5% CO_2_. To determine cell viability, MTT 50 mg/mL in PBS was added to each well and incubated for 4 h at 37 °C. Finally, 100 μL of 2-propanol/1M HCl (19:1 *v*/*v*) was added to dissolve the formazan crystals. Absorbance measurements were conducted utilizing a microplate spectrophotometer reader (BioTek Instruments, Winooski, VT, USA) at 595 nm.

### 4.6. Necrosis and Apoptosis Assay

HeLa cell line was seeded in 96-well flat-bottomed plates at a density of 3 × 10^4^ cells per well in 200 μL of CM and incubated for 24 h at 37 °C with 5% CO_2_. Then, cells were synchronized with SS medium for 12 h under the same conditions and adding different concentrations of CDPs. DMSO was used as control at the same concentration used to dissolve the CDPs. To determinate the apoptotic effect, cells were collected by centrifugation at 2000× *g* for 10 min. The pellet was suspended in 20 μL of SS medium and treated with annexin V and propidium iodide (PI) (Dead Cell Apoptosis Kit; Molecular Probes, Invitrogen Life Technologies), or with 7-aminoactinomycin D (7-AAD; Molecular Probes, Invitrogen Life Technologies) following the indications recommended by the manufacturer. Fluorescence was immediately quantified by flow cytometry using an Accuri-C6 Flow Cytometer (BD Biosciences, San Jose, CA, USA). Cell populations from each treatment were gated in forward scatter and side scatter dot plots to eliminate cell debris. Populations corresponding to auto- or basal-fluorescence were located in the left quadrant, and cells with emission of fluorescence increasing at least one log unit value were located in the right quadrant of the dot plots. In addition, the percentage of fluorescent cells (PFC) and median fluorescence intensity (FI) were determined in monoparametric histograms of fluorescence emission obtained from the dot plots and labeled as PFC and as relative fluorescence units. The equipment was calibrated using Spherotech 8-peak (FL1-FL3) and 6-peak (FL-4) validation beads (BD Accuri, San Jose, CA, USA). For apoptosis and necrosis assays, fluorescence for annexin V in emission fluorescence channel FL1 at 495/519 nm, for propidium iodide in the FL2 channel at 535/617 nm, and for 7-AAD in the FL3 channel at 488/647 nm were monitored. At least 20,000 cellular events were analyzed for each determination point. Data were analyzed using FlowJo V12.1 software (Tree stat, Stanford, CA, USA).

### 4.7. Caspases Inhibition Assays

HeLa cell line was seeded 2 × 10^5^ cells per well in 24 flat bottom plates in 0.5 mL of CM medium. Cells were syncronized for 12 h in SS medium and after the caspases inhibitors: pan caspase (Z-VAD-FMK), caspase-3 inhibitor (Z-DEVD-FMK), caspase-8 inhibitor (Z-IETD-FMK), and caspase-9 inhibitor (Z-LEHTD-FMK) (BD Pharmigen, San Jose, CA, USA) at 10 mM concentration were added 120 min prior to the addition of crude PAO1-CDPs mix at 10 mg/mL, followed by 4 h of incubation. DMSO was used as negative control in absence of caspase inhibitor in the same condition as the crude PAO1-CDPs mix. Cells were collected by trypsinization, and washed with cold PBS. Apoptosis was monitored using anexin V-APC (allophycocyanin) conjugated (BD Pharmigen) and fluorescence was registered in Accuri-C6 flow cytometer by fluorescence emission (650/660 nm) determined in FL4 channel. At least 20,000 cellular events were analyzed in each determination point, data after were analyzed using FlowJo V12.1 software.

### 4.8. Determination of IL-8 by ELISA

HEK-293 TLR4/MD2/CD14 cell line that stably expressed TLR4 receptor were seeded into a 96-well plate at a concentration of 2 × 10^4^ cells per well and treated with the crude PAO1-CDPs mix for 4 h. Cell supernatants were tested for IL-8 protein with the commercially available OptEIATM kit (BD Biosciences), absorbance was measure at 490 nm in ELISA reader (Dynex, Chantilly, VA, USA).

### 4.9. Mitochondrial Membrane Potential Determination

Membrane potential in HeLa cells suspension was determined using the fluorescent, cell-permeable indicator Rhodamine 123 (Sigma-Aldrich Co.). HeLa cell line was seeded in 96-well flat-bottomed plates at a density of 3 × 10^4^ cells per well in 200 μL of CM and incubated for 24 h at 37 °C with 5% CO_2_. Then, cells were synchronized with SS medium for 2 h under the same conditions and adding 0.1 mg/mL of the crude PAO1-CDPs mix. DMSO was used as control at the same concentration used to dissolve the CDPs. After, cells were loaded with Rhodamine 123 (5 µg/mL) and incubated at 37 °C for 30 min in darkness. Suspensions were washed and fluorescence was quantified using an Accuri-C6 Flow Cytometer monitoring the emission fluorescence in channel FL1 at 533/30 nm. At least 20,000 cellular events were analyze; or directly observed in a Confocal Microscopy (FV1000, Olympus, Center Valley, PA, USA) monitoring the emission fluorescence at 533/30 nm. Fluorescence intensity was quantified using the Image J software.

### 4.10. Real-Time Quantification of Superoxide in Human Tumor Cell Lines

Intracellular superoxide (O_2_^•−^) in cell suspensions was determined using cell-permeant fluorescent probe dihydroethidium (DHE, Molecular Probes, Invitrogen) and fluorescence was quantified by flow cytometry using an Accuri-C6 Flow Cytometer. Human cell lines were grown as described above and samples (100 µL) were trypsinized and washed with PBS buffer. Cells suspensions (1 × 10^5^ cells) were incubated with DHE (5 µg/mL) at 37 °C for 2 h in darkness. Then, human cells were harvested, washed, and re-suspended in PBS. The populations of fluorescent cells for each treatment were monitored by flow cytometry in the emission fluorescence channel FL1 (587/40 nm). At least 20,000 cellular events were analyzed in each determination point.

### 4.11. Immunodetection Assays

Human HeLa cell cultures were grown as described above and synchronized by 12 h in incomplete medium without serum (SS) incubating at 37 °C under 5% CO_2_ atmosphere. 3 × 10^4^ cells were seeded in each well (six-well plates) in total volume per well of 3 mL of fresh SS or CM mediums supplemented with respective compounds to test. After treatments, the medium was eliminated and cells were submitted to cellular trypsinization with CM/SS medium and harvested by centrifugation at 5000× *g*, 4 °C by 10 min. Cellular lysis was carried out in phosphorylation buffer (PB) 300 µL composed by [Hepes 50 mM pH 7.6, sodium-pyrophosphate 50 mM, sodium ortovanadate 1 mM, sodium molybdate 1 mM, EDTA, EGTA 20 mM, benzamidine 1 mM, NaF 20 mM, PMSF 0.2 mM, ß-glycerophosphate 80 mM, mannitol 200 mM, protease inhibitor cocktails 1 µL/mL (all reagents from Sigma-Aldrich Co.)]. Cell suspension was lysed (cell lysate) by two sonication pulses at low intensity by 30 sec each at 4 °C (Hielscher-LS24 Utrasound Technol., Ringwood, NJ, USA). The protein extracts cell-free were obtaining by centrifugation of total cell homogenates at 7500× *g*, 4 °C by 15 min. Protein was determined by Bradford method (BioRad, Hercules, CA, USA) and 30 µg of total protein was mixed with 10 µL of denaturing buffer (Tris-HCl 0.06M, pH6.8, 5% de glycerol, 4% SDS, 4% β-mercaptoethanol and 0.0025% bromophenol blue) during 5 min at 95 °C in a boiling water bath. Samples were run in a denaturing polyacrylamide gel electrophoresis at 10–12% (SDS-PAGE). The gels in one side were Coomassie blue stained and the other gel transferred to polyvinylidene difluoride (PVDF, Millipore, Billerica, MA, USA) membranes for western blot procedure.

For immunodetection, membranes were blocked using dry milk in TBS-T (Tris-HCL 10 mM; NaCl 0.9%; tween-20 0.1%, pH 7.8) and blotted with the anti-human antibodies: anti-Akt (C-20-R), anti-Akt-phosphoryled 1/2/3 (Ser 473-R), anti-p70 S6 kinase α (H-160), anti-phosphoryled-p70 S6 kinase α (Thr 389)-R, and anti-ß-actin; all from Santa Cruz Biotechnology, Santa Cruz, CA, USA. The first antibody was blotted in blocking medium at 1:10,000 dilution for 12 h at 4 °C with light shaking. After washing, the membrane was incubated with the secondary antibody, Goat anti-Rabbit IgG HRP-conjugate (BioRad), in blocking medium at 1:10,000 dilution for 4 h at 4 °C; the membrane was twice washed with TBS-T and developed using hydrogen peroxide and Supersignal West Pico Luminol (Pierce, Thermo Fisher Scientific) and after exposing in light-sensitive films or ChemiDoc™ MP System (Bio-Rad). Assays were conducted by at least three independent assays and representative images are shown. Bands intensities in gels or films were quantified using the Image J1 software (NIH Image, Bethesda, MA, USA).

### 4.12. Cell Image Captures

HeLa cells was seeded in 12-well flat-bottomed plates at a density of 1 × 10^4^ cells per well with 1 mL of CM and incubated for 24 h at 37 °C with 5% CO_2_. Cells were incubated with serum-free medium (SS) for 12 h at 37 °C and an atmosphere of 5% CO_2_ and incubated with different concentrations of the CDPs. After treatment, the cells were washed with PBS. Cells were fixed with paraformaldehyde (PFA at 4%) for 10 min on ice and collocated on cover glass, placed into a holder with a drop of PBS and glycerol 1:1 and photographed using an inverted phase-contrast microscope (Carl-Zeiss HB0-50, Gottingen, Germany) equipped with an AxioCam/Cc1 digital camera (Carl-Zeiss, Gottingen, Germany). Additionally cell cultures were observed directly using a confocal microscope (Olympus FV1000), images of the HeLa cells were taken using 40× magnification.

### 4.13. Ethical Considerations

The Hospital Juarez of Mexico Scientific Research Committee (composed of Scientific, Ethics, and Bio-security Committees) approved the project (projects number: HJM 2321/14B, HJM2112/12-B), and in accordance with “Reglamento de la Ley General de Salud en Materia de Investigación para la Salud, Mexico”, and the protocols that were used conformed to the ethical guidelines of the 1975 Declaration of Helsinki. All enrolled individuals provided written informed consent.

## 5. Conclusions

Our findings indicate that the antiproliferative effect of the PAO1-CDP mixture on HeLa cells involves inhibition of both Akt-S473 and S6k-T389 protein phosphorylation and activation of the caspase-9-dependent intrinsic apoptosis pathway and mitochondrial dysfunction. These data suggest that the antiproliferative effect of PAO1-CDPs involves the Akt–mTOR–S6k signaling pathway, pointing to the involvement of mTORC complexes.

## Figures and Tables

**Table 1:** Induction of cellular dead and apoptosis on human tumor HeLa cells by the *P. aeruginosa* PAO1 cyclodipeptides.

Cyclodipeptide	Viability LD_50_ (mg/mL) ^1^	Apoptosis EC_50_ (mg/mL) ^1^
PAO1-Cyclo(l-Pro-l-Tyr)	0.061/0.053	3.2 × 10^−4^/1.6 × 10^−4^
PAO1-Cyclo(l-Pro-l-Val)	0.037/0.035	2.7 × 10^−4^/2.8 × 10^−4^
PAO1-Cyclo(l-Pro-l-Phe)	0.015/0.010	5.0 × 10^−4^/4.1 × 10^−4^
Crude PAO1-CDPs mix	0.060/0.062	4.9 × 10^−4^/2.3 × 10^−4^
Synthetic cyclo(l-Pro-l-Tyr)	10.7/11.4	ND
Synthetic cyclo(l-Pro-l-Val)	19.3/60.7	ND
Synthetic cyclo(l-Pro-l-Phe)	90.9/154	ND
Synthetic CDPs mix	2.90/2.04	100

^1^ LD_50_: Half lethal doses and EC_50_: half effective concentration determined after 24 h of treatment with CDPs, being calculated using Nonlinear regression (curve fit), log(inhibitor/ inductor) vs response (variable slop), R^2^ = 0.95–0.99 (GraphPad Prism 5.0). ND, not determined. Data obtained using: complete medium/incomplete medium.

## Supplementary Materials

Supplementary materials are available online.
