# Supplementary material for: The Antiproliferative Effect of Cyclodipeptides from Pseudomonas aeruginosa PAO1 on HeLa Cells Involves Inhibition of Phosphorylation of Akt and S6k Kinases
Source: Molecules. 2017 Jun 20;22(6):1024. doi: 10.3390/molecules22061024 (PMC6152764; doi:10.3390/molecules22061024)
Supplement: Supplementary file 1 [file molecules-22-01024-s001.pdf]

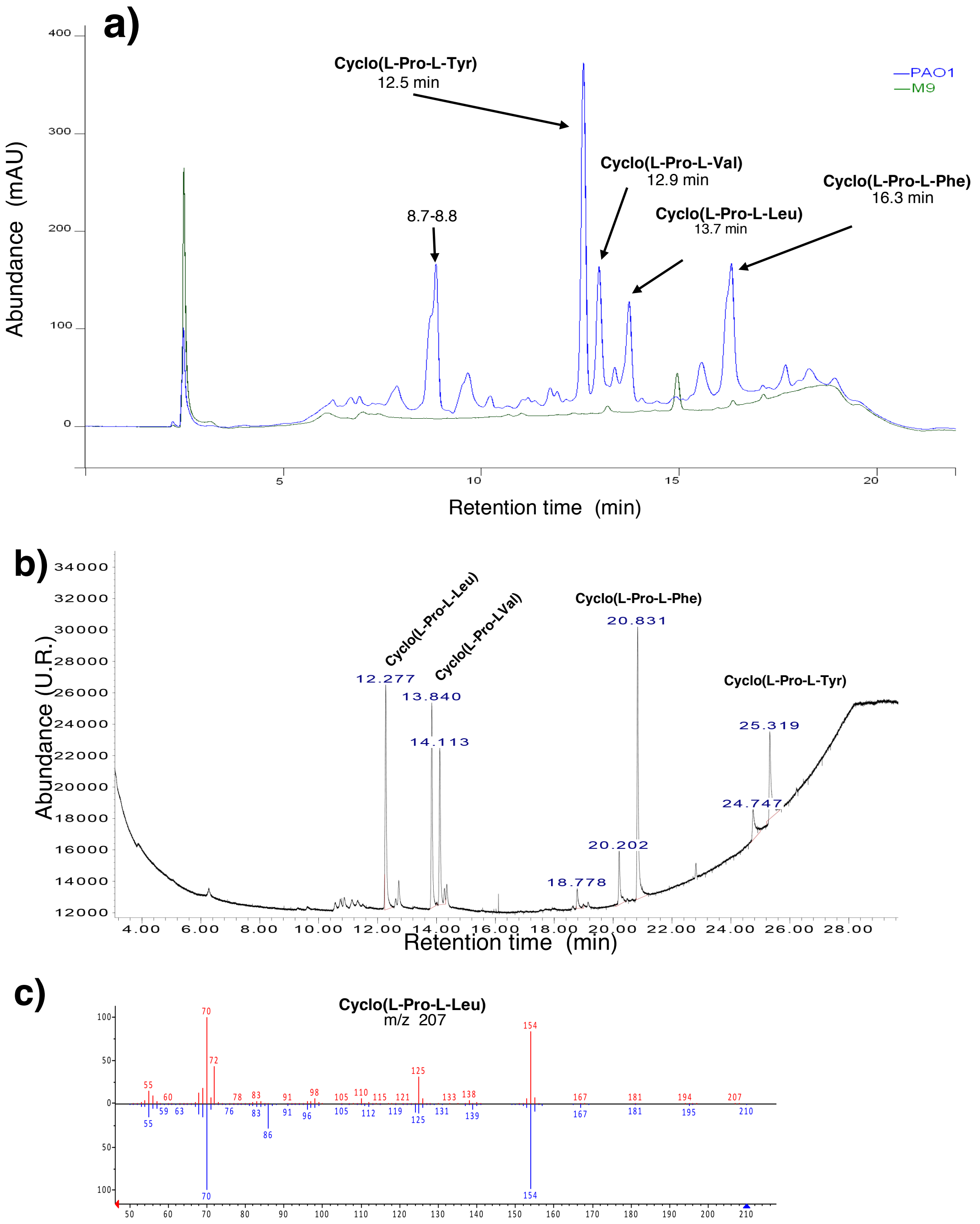
a)
Cyclo(L-Pro-L-Tyr)
12.5 min
Cyclo(L-Pro-L-Val)
12.9 min
Cyclo(L-Pro-L-Leu)
13.7 min
Cyclo(L-Pro-L-Phe)
16.3 min
8.7-8.8
—PAO1
—M9
Abundance (mAU)
Retention time (min)
b)
Cyclo(L-Pro-L-Leu)
Cyclo(L-Pro-LVal)
Cyclo(L-Pro-L-Phe)
Cyclo(L-Pro-L-Tyr)
12.277
13.840
14.113
18.778
20.202
20.831
24.747
25.319
Abundance (U.R.)
Retention time (min)
c)
Cyclo(L-Pro-L-Leu)
m/z 207

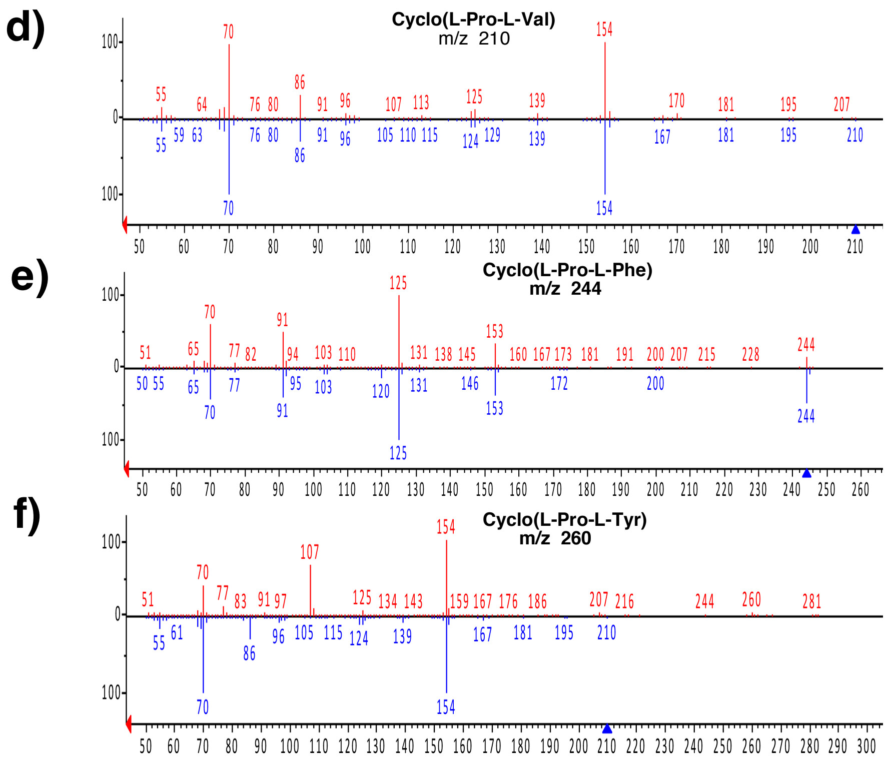

Figure S1. Chromatographic profiles of the CDPs mix produced by *P. aeruginosa* PAO1. CDPs-extract was obtained from supernatant of PAO1 cultures extracted twice with ethyl acetate as described in Materials and Methods. The extract was dissolved in 1 mL in HPLC-grade acetonitrile. Analysis of extract was carried out using High Performance Liquid Chromatography (HPLC) using a Photo Diode Array detector and a reverse-phase HPLC column Sephasil-Peptide C18, 12μ 4.6 mm × 250 mm (Amersham). **(a)** HPLC profile of the crude PAO1-CDPs extract. The peaks marked as the CDPs cyclo(L-Pro-L-Tyr), cyclo(L-Pro-L-Val), and cyclo(L-Pro-L-Phe) were collected and after utilized for viability and apoptosis assays on HeLa cell line. The fractions were analyzed by GC-MS for compound identification **(b)** as described in Materials and Methods. **(c-f)** CDPs fragmentation profiles were compared with the respective synthetic CDPs (Bachem Co.). The arrows indicate the fractions corresponding to CDPs, whose mass fragmentation profiles are showed, (red profile) obtained in this work and (blue) obtained from the NIST library, with ≥ 90% of identity.
